# Troponin and a Myopathy-Linked Mutation in *TPM3* Inhibit Cofilin-2-Induced Thin Filament Depolymerization

**DOI:** 10.3390/ijms242216457

**Published:** 2023-11-17

**Authors:** Katarzyna Robaszkiewicz, Julia Wróbel, Joanna Moraczewska

**Affiliations:** Department of Biochemistry and Cell Biology, Faculty of Biological Sciences, Kazimierz Wielki University in Bydgoszcz, 85-671 Bydgoszcz, Poland; robkat@ukw.edu.pl (K.R.); wrobel.julia1998@gmail.com (J.W.)

**Keywords:** thin filament, tropomyosin, myopathy, troponin, cofilin-2, actin

## Abstract

Uniform actin filament length is required for synchronized contraction of skeletal muscle. In myopathies linked to mutations in tropomyosin (Tpm) genes, irregular thin filaments are a common feature, which may result from defects in length maintenance mechanisms. The current work investigated the effects of the myopathy-causing p.R91C variant in Tpm3.12, a tropomyosin isoform expressed in slow-twitch muscle fibers, on the regulation of actin severing and depolymerization by cofilin-2. The affinity of cofilin-2 for F-actin was not significantly changed by either Tpm3.12 or Tpm3.12-R91C, though it increased two-fold in the presence of troponin (without Ca^2+^). Saturation of the filament with cofilin-2 removed both Tpm variants from the filament, although Tpm3.12-R91C was more resistant. In the presence of troponin (±Ca^2+^), Tpm remained on the filament, even at high cofilin-2 concentrations. Both Tpm3.12 variants inhibited filament severing and depolymerization by cofilin-2. However, the inhibition was more efficient in the presence of Tpm3.12-R91C, indicating that the pathogenic variant impaired cofilin-2-dependent actin filament turnover. Troponin (±Ca^2+^) further inhibited but did not completely stop cofilin-2-dependent actin severing and depolymerization.

## 1. Introduction

Congenital myopathies are heterogeneous neuromuscular disorders caused by mutations in about 50 genes encoding sarcomeric proteins. Functional abnormalities characterizing various types of myopathies include progressive muscle weakness, changes in sarcomere structure, and defects in actin–myosin interaction at the physiological and biochemical levels [1,2,3,4]. Numerous missense mutations in *TPM3*, the gene encoding the muscle tropomyosin (Tpm) isoform Tpm3.12, have been associated with nemaline myopathy, cap disease, and congenital fiber-type disproportion [5,6,7]. At the molecular level, the typical phenotype associated with the mutations is hypocontractility, characterized by reduced activation of actin–myosin ATPase and lower sensitivity of ATPase to the activating Ca^2+^ concentrations [8,9,10]. Hypocontractility correlates with muscle weakness observed in affected humans and is a direct consequence of distortions in the regulatory functions of Tpm3.12 [6,11].

The force generated by muscle fibers depends on the efficiency of actin–myosin [12]. We have previously shown that myopathy-causing mutations in Tpm3.12 affect length regulation by tropomodulin, a pointed [13]. Thus, thin filament length aberrations may be another hallmark of the myopathic phenotype.

Tpm is an α-helical dimer forming a coiled-coil rod that polymerizes end-to-end along the length of the actin filament. The contractile thin filaments in skeletal muscle are associated with three different Tpm isoforms: Tpm1.1, Tpm2.2, and Tpm3.12. The latter is specific to mature slow-twitch muscle fibers, where it replaces Tpm1.1 [14,15]. In striated muscle, each Tpm binds the Ca^2+^-binding troponin (Tn) complex to regulate actin–myosin interactions in response to muscle activation and relaxation [16]. According to the classical steric-blocking mechanism, the Tpm-Tn complex can assume various azimuthal positions on the thin filament [17,18]. Besides regulating actin–myosin interactions, the activation states of the thin filament can be important for the regulation of actin interactions with proteins that determine the filament’s length and turnover, processes important for thin filament integrity. In our recent work, we demonstrated that skeletal muscle isoforms Tpm1.1 and Tpm3.12 have different capacities to regulate the severing and depolymerization activities of cofilin-2 [19]. However, it is not known how the presence of Tn on the filament and different activation states produced by Ca^2+^ binding to Tn affect interactions of cofilin-2 with actin. This problem needs to be addressed because Tn is a key component of the thin filament regulatory machinery in striated muscle.

Cofilin-2 is a muscle-specific member of ADF/cofilin, a family of actin depolymerizing factors that are crucial for actin filament dynamics and maintenance of muscle homeostasis [20,21]. In striated muscle, cofilin-2 is mostly located near the pointed end of the thin filaments that are directed towards the center of the sarcomere [22]. The importance of cofilin-2 for skeletal muscle maintenance was demonstrated in *CFL2*-knockout mice, which displayed sarcomere disruption, degeneration of the myofibrillar apparatus, and excessive accumulation and aggregation of actin filaments in muscle [23,24,25]. In humans, missense mutations in *CFL2* are associated with nemaline myopathy and myofibrillar myopathy [26,27,28], with loss of cofilin-2 functions resulting in sarcomeric and cytoskeletal disorders. Although it is well established that Tpm regulates cofilin-2 [19,20,22], it is not known whether myopathy-causing mutations affect its activity.

The primary cause of muscle weakness observed in patients with congenital myopathies linked to single amino acid substitutions in Tpm3.12 is decreased activation of the actomyosin interactions by Ca^2+^ [6,29,30], but defects in thin filament length maintenance may also affect contraction and, through this mechanism, contribute to the myopathic phenotype [12,13,31]. Since knowledge of the effects of Tpm3.12 variants on the filament dynamics is limited, the goal of this work was to verify the hypothesis that through affecting cofilin-2 activity, Tn and myopathy-causing Tpm3.12 variants influence length maintenance and stability. To address this problem, we assessed whether the presence of Tn and the actin filament activation state affected cofilin-2 binding to the thin filament and its efficiency in severing and depolymerizing actin. We then analyzed how the myopathy-causing Tpm3.12 variant changes the interactions of cofilin-2 with actin filament. Analyses were performed in vitro using thin filaments reconstituted from muscle actin, Tn, and recombinant wild-type or mutant Tpm3.12 variants.

## 2. Results

### 2.1. Effects of the p.R91C Mutation in Tpm3.12 and the Troponin Complex on Cofilin-2 Binding to F-Actin

To gain insight into the regulation of cofilin-2 activity by a myopathy-causing mutation in *TPM3*, we used the p.R91C substitution in Tpm3.12, which is associated with muscle weakness in patients [5] and causes the hypocontractile phenotype in vitro [13].

**Figure 1:**
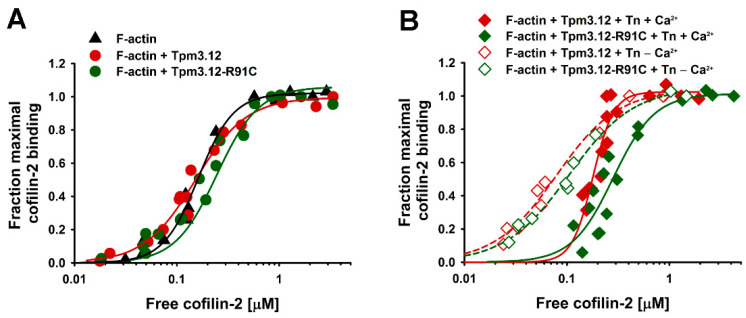
Binding of cofilin-2 to F-actin regulated either by Tpm3.12 or Tpm3.12-R91C in the absence (**A**) or presence of Tn ± Ca^2+^ (**B**). The experimental points were fit to the Hill model. Conditions: 3.0 μM F-actin saturated with 2 μM Tpm3.12 or Tpm3.12-R91C ± 3 μM Tn, and cofilin-2 at concentrations ranging from 0 to 10 μM in 5.0 mM Tris–HCl, pH 7.5, 2 mM MgCl_2_, 100 mM NaCl, ±0.1 mM CaCl_2_. The data were collected from two independent experiments.

Cofilin-2 binding to the thin filament was analyzed using a cosedimentation assay, which confirmed previous observations [21,32] that unphosphorylated recombinant cofilin-2 binds strongly to unregulated F-actin. The presence of Tpm3.12 had no significant effect on cofilin-2 affinity but reduced binding cooperativity. The effect of the p.R91C mutation was mild, causing a slight decrease in the apparent actin-binding constant (K_app_) and no significant change in binding cooperativity compared to wild-type Tpm3.12 (Figure 1A and Table 1).

Since the regulatory proteins of the thin filament in the muscle cells consist of the Tpm-Tn complex, in the next step, we analyzed cofilin-2 binding to the thin filament in the presence of Tn (±Ca^2+^). Interestingly, interactions of cofilin-2 with actin were strongly affected by the activation state of the thin filament. Compared with Tpm3.12 alone, the presence of Tpm3.12 and Tn complex saturated with Ca^2+^ caused a small K_app_ decrease, but removal of Ca^2+^ from Tn led to a two-fold increase in cofilin-2 affinity for actin (Figure 1B). In the presence of the Tpm3.12-R91C variant, cofilin-2 tended to bind the thin filament more weakly, but when compared with the wild-type Tpm3.12, the difference in K_app_ was statistically insignificant (Table 1).

### 2.2. Effects of the Troponin Complex on Dissociation of Tpm3.12 and Tpm3.12-R91C from Actin Filaments

**Figure 2:**
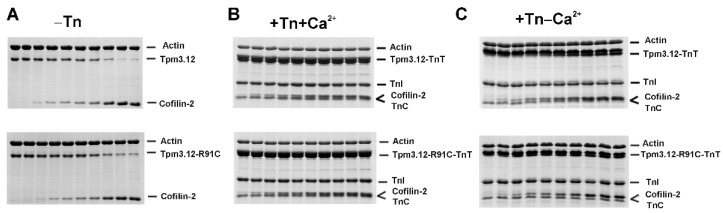
Electrophoretic separation of the proteins collected in pellets after ultracentrifugation of the regulated thin filaments incubated with increasing concentrations of cofilin-2. (**A**) F-actin coated with Tpm3.12 (upper panel) or Tpm3.12-R91C (lower panel). (**B**) F-actin coated with Tpm3.12 (upper panel) or Tpm3.12-R91C (lower panel) in the presence of Tn + Ca^2+^. (**C**) F-actin coated with Tpm3.12 (upper panel) or Tpm3.12-R91C (lower panel) in the presence of Tn − Ca^2+^. Conditions: 3 μM F-actin, 2 μM Tpm3.12 or Tpm3.12-R91C, 3.0 μM Tn, 0.1 mM CaCl_2_ or 0.2 mM EGTA, and cofilin-2 at concentrations ranging from 0 to 10 μM in 5.0 mM Tris–HCl, pH 7.5, 2 mM MgCl_2_, 100 mM NaCl.

In the absence of Tn, the increasing occupation of the actin filament with cofilin-2 causes dissociation of various Tpm isoforms from the thin filament [19,32], which is consistent with the perception of Tpm and cofilin as competitors [33]. The ability of cofilin-2 to eject Tpm3.12 variants from the filament was assessed by densitometric analysis of the SDS gels used to visualize proteins pelleted with F-actin at increasing cofilin-2 concentrations (Figure 2).

**Figure 3:**
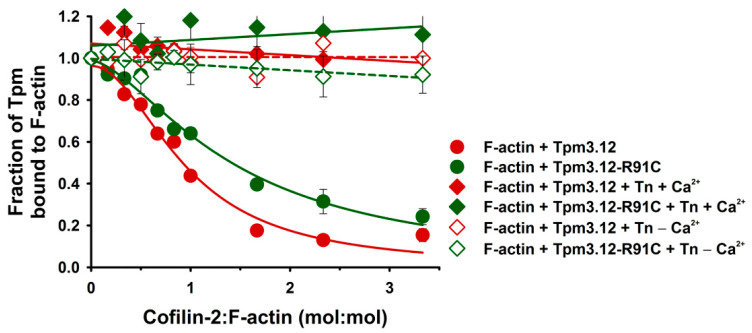
Cofilin-induced dissociation of Tpm3.12 and Tpm3.12-R91C from F-actin in the absence and presence of Tn ± Ca^2+^. In the absence of Tn, the averaged experimental points were fit to decay regression. In the presence of Tn ± Ca^2+^, the averaged experimental points were fit to linear regression. Conditions: 3.0 μM F-actin saturated with 2 μM Tpm variants, 3 μM Tn ± Ca^2+^, 5.0 mM Tris–HCl, pH 7.5, 2 mM MgCl_2_, 100 mM NaCl, and 0.1mM CaCl_2_ or 0.2 mM EGTA were titrated with cofilin-2 at concentrations ranging from 0 to 10 μM. The points are means of three independent experiments ± SE.

Cofilin-2 removed Tpm3.12 from 3 μM F-actin with a half-maximal inhibitory concentration (IC_50_ = 0.75 ± 0.04), which is in excellent agreement with previous data [19]. The p.R91C mutation had a stabilizing effect, significantly reducing the cofilin-2 ability to remove Tpm3.12-R91C from actin and shifting the IC_50_ toward a higher cofilin-2 concentration (IC_50_ = 1.45 ± 0.07) (Figure 3). The differences in IC_50_ in the presence of Tpm3.12 and Tpm3.12-R91C were statistically significant (*p* < 0.05).

Next, we assessed the capacity of cofilin to eject Tpm and Tn from the filament. Surprisingly, the presence of Tn strongly stabilized Tpm3.12 and Tpm3.12-R91C on the filament, regardless of the presence or absence of Ca^2+^ (Figure 3). The representative SDS gels shown in Figure 2 revealed that all components of the thin filament were present in the pellets, even at a three-fold molar excess of cofilin-2 over actin. Since Tpm and TnT bands were not well separated (Figure 2), the experimental points illustrated in Figure 3 show Tpm-TnT fractions bound to F-actin, which was stable for both variants of Tpm3.12 in two activation states (±Ca^2+^) within the range of cofilin-2 concentrations used in the experiment.

### 2.3. Effects of Tpm3.12 Variants and Troponin on Cofilin-2-Induced Fragmentation and Depolymerization of F-Actin

**Figure 4:**
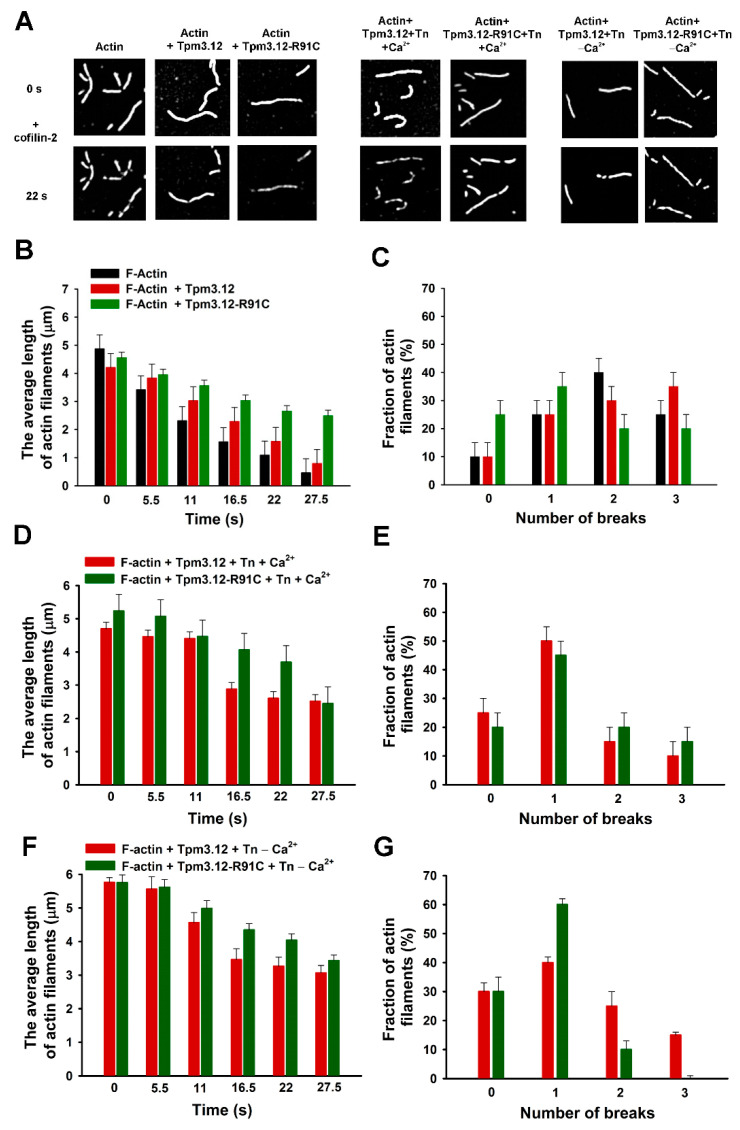
The effects of Tpm3.12, Tpm312-R91C, and Tn on cofilin-2-induced filament severing and depolymerization. Snapshots of F-actin alone and F-actin regulated by Tpm3.12 or Tpm3.12-R91C in the presence of Tn and Ca^2+^ before (0 s) and after (22 s) the addition of cofilin-2 (**A**). The mean length of F-actin, F-actin regulated by Tpm3.12 or Tpm3.12-R91C (left panel), and the fraction of filaments with breaks (right panel) in the absence of Tn (**B**,**C**), in the presence of Tn + Ca^2+^ (**D**,**E**), and the presence of Tn +Ca^2+^ (**F**,**G**). Conditions: 25 mM Tris-HCl, pH 7.5, 25 mM KCl, 2 mM MgCl_2_, and 10 mg/mL of BSA supplemented with 0.1 mg/mL of glucose oxidase, 0.01 mg/mL of catalase, and 3 mg/mL of glucose. Error bars are standard error (SE) calculated from 50 filaments. A comparison of the differences between the experimental groups showed that the differences were significant in: (**B**) at the time range from 11 to 27.5 s (*p* < 0.05); (**C**) within groups with different numbers of breaks (*p* < 0.05); (**D**,**F**) at the time range from 16 to 27.5 sec (*p* < 0.05); (**E**,**G**) within groups with different numbers of breaks (*p* < 0.05).

Fluorescence microscopy was used to investigate the role of actin regulatory proteins on cofilin-2-dependent severing and depolymerization. Actin filaments were loosely immobilized on the surface of a positively charged lipid bilayer [34] The use of a lipid bilayer instead of myosin heads (HMM), which were applied in the previous work [19], allowed us to obtain stable, unbroken filaments before introducing cofilin-2 to the flow cells. Cofilin-induced changes in the length and number of breaks in individual filaments were followed for up to 30 s after adding cofilin-2 (Figure 4A).

The depolymerization activity of cofilin-2 was assessed by measuring, at time intervals, the length of the longest filament fragments remaining after severing the filament by cofilin-2. The presence of both Tpm3.12 variants protected the filament against depolymerization by cofilin-2, but Tpm3.12-R91C-coated filaments were more resistant to the depolymerizing effects of cofilin-2 than the wild type (Figure 4B, left panel).

Exposing the unregulated and regulated actin filaments to cofilin-2 for 22 s resulted in the formation of one to three breaks per filament. The severing activity of cofilin-2 appears to be inhibited by the mutation p.R91C, as in the presence of Tpm3.12-R91C, approximately 60% of the filaments had zero to break, while only 35% of the filaments had zero to one break in the presence of wild-type Tpm3.12 (Figure 4B, right panel).

**Figure 5:**
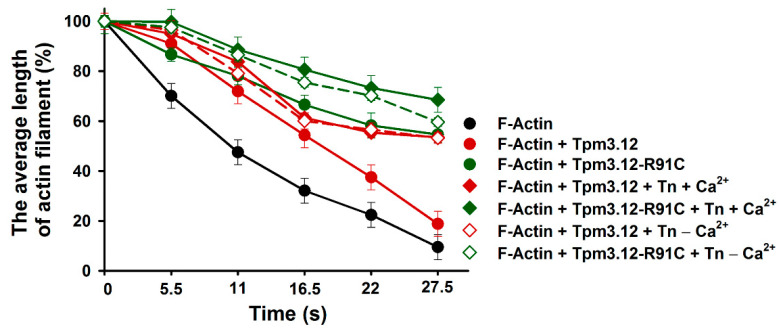
The rate of actin filament depolymerization in the presence of Tpm variants and Tn ± Ca^2+^. The lengths of the filaments at different time points are shown as the percentage of the lengths obtained before adding cofilin-2. The conditions were the same as in Figure 4. The differences were statistically significant between F-actin and F-actin-Tpm3.12 or F-actin-Tpm3.12-R91C within the whole time range (*p* < 0.05). The difference between F-actin-Tpm3.12 and F-actin-Tpm3.12-R91C was significant at the time range of 16.5 to 27.5 s (*p* < 0.05). The differences between F-actin-Tpm3.12 and Tpm3.12-R91C in the presence of Tn (±Ca^2+^) were significant at the time range of 16.5 to 27.5 s (*p* < 0.05).

Next, we assessed whether Tn (±Ca^2+^) affected cofilin-2-induced thin filament depolymerization and severing. Since the Tn complex strongly stabilized Tpm3.12 on the filament by preventing the dissociation of Tpm from actin (Figure 2 and Figure 3), one could expect that Tn would protect the filament against cofilin-2-dependent depolymerization and severing. Surprisingly, the obtained results showed that cofilin-2 still depolymerized and severed the filament (Figure 4C,D, left panels). To compare depolymerization rates in the presence of different combinations of regulatory proteins, filament lengths obtained at the selected time points were normalized by dividing the mean filament length by the values obtained in the absence of cofilin-2 (time 0), with shortening of the filament plotted as a function of time (Figure 5). The depolymerization rates showed inhibitory effects of both Tpm3.12 variants, which was further enhanced by Tn, while the p.R91C mutation decreased the depolymerization rate in the presence of Tn. However, the activation state of the filament determined by Ca^2+^ binding to the Tn complex did not affect depolymerization. Along with decreasing the depolymerization rate, Tn (±Ca^2+^) reduced the thin filament severing. In both activation states, the dominating filament fractions (70–90%) had zero to one break. The Tn- Tpm3.12-R91C complex did not induce three or more breaks, demonstrating that that it had a stronger tendency to inhibit severing than the wild-type Tn-Tpm3.12 complex (Figure 4D, right panels).

## 3. Discussion

The classical function of the Tpm-Tn complex is regulation of contraction by controlling actin–myosin interactions in a Ca^2+^-dependent manner [16]. Since thin filaments in the sarcomere interact not only with myosin but also with proteins that regulate the length [33,35,36], the Tpm-Tn complex may also be involved in regulating actin length maintenance. Dysregulation of this process may contribute to the pathogenesis of congenital myopathies linked to mutations in the proteins of the thin filament. In this work, we analyzed how the p.R91C variant of Tpm3.12 found in myopathy patients affects the in vitro activity of cofilin-2, the muscle-specific isoform of the ADF/cofilin family of proteins that maintain their length through severing and depolymerization of actin filaments.

### 3.1. Effects of Troponin and Tpm3.12-R91C on Actin Affinity of Cofilin-2

Tpm isoforms specifically expressed in skeletal muscle fibers have a very mild effect, if any, on the K_app_ of cofilin-2 [19,22,32], which implies that the binding sites on actin for these two proteins do not overlap. By binding to F-actin, cofilin bridges two adjacent actin subunits along the filament, with the region of cofilin that binds to both the actin filament and the monomer (G-site) making extensive contacts with actin subdomain 1 and the bottom of subdomain 3. Meanwhile, the F-site binds to the lower actin subunit and forms an interface with actin subdomain 1 [37,38,39]. A high-resolution structure of cofilin-2 bound to F-actin-Tpm is not available, but a recent structure of cofilin-2 decorating naked F-actin has determined the details of the cofilin-2–actin interface [39]. The actin residues involved in interactions with cofilin-2 do not appear to overlap with those that form the interface with Tpm, except for R28, which interacts with both Tpm and cofilin-2. Therefore, the negligible effect of wild-type Tpm3.12 on the actin affinity of cofilin-2 is in good agreement with these findings. The p.R91C myopathy-causing mutation neutralizes the charge of the actin-binding residue and may therefore lead to subtle conformational changes in actin, causing reduced affinity. It is most likely that the decreased affinity of cofilin-2 for F-actin decorated with Tpm-R91C is responsible for its impaired ability to remove Tpm3.12-R91C from actin.

In skeletal muscle fibers, the Tn complex is closely associated with the thin filament, so its presence should be taken into account in the analysis of cofilin-2 binding to actin. The data collected in the present study showed that in the Ca^2+^-bound state, Tn did not significantly change the affinity of cofilin-2 for actin, while it increased the affinity approximately two-fold in the Ca^2+^-free state. We also found that Tn stabilized Tpm3.12 on the filament. Unlike Tpm alone (this work and [19]), the Tpm3.12-Tn (±Ca^2+^) complex was not removed by increasing filament occupation with cofilin-2, suggesting that the Tpm3.12-Tn complex can be accommodated on F-actin along with cofilin-2 in both activation states of the filament. In a previous study [34], we suggested that removing Tpm by increasing the occupation of actin by cofilin is caused by the cofilin-induced twist of the filament [40] that disrupts the Tpm–actin interface. Does this mean that the filament is not twisted by cofilin-2 in the presence of Tn? There is currently no answer to this question. Due to the lack of high-resolution structures of the complex of regulated thin filaments with cofilin, the structural explanation for the simultaneous binding of cofilin, Tpm, and Tn in the presence and absence of Ca^2+^ is not available.

### 3.2. Effects of Troponin and Tpm3.12-R91C on Severing and Depolymerization of the Thin Filament by Cofilin-2

Although binding of cofilin-2 to the F-actin-Tpm removes Tpm from the filament, the initial presence of Tpm on the filament inhibits severing and depolymerization of the severed filament fragments [32,41,42]. Previously, we have shown that sequence differences between the muscle isoforms of Tpm affect their abilities to inhibit cofilin activities [19,32]. In this work, we demonstrated that exchanging even one amino acid in Tpm3.12 can disturb the regulation of cofilin-2 activities. The severing and depolymerization assay revealed that the p.R91C substitution in Tpm3.12 reduced the number of breaks introduced by cofilin-2 and inhibited depolymerization of the filament. This correlated with reduced affinity of cofilin-2 to the filament decorated by Tpm3.12-R91C, which can be caused by partial blocking of cofilin-2 binding sites on actin due to a shift of Tpm3.12-R91C chains on the filament. Alternatively, decreased affinity of cofilin-2 could be caused by conformational change in the actin–cofilin interface induced by the presence of Tpm3.12-R91C.

The Tn complex had a stabilizing effect by reducing the number of gaps produced by cofilin-2 and slowing down the depolymerization rate. Interestingly, although all proteins bound to the filament, neither severing nor depolymerization was completely blocked, which, to our knowledge, is a novel observation. Since Ca^2+^ binding causes an azimuthal shift of Tpm chains on the filament [17,43], one can hypothesize that Tpm regulates cofilin activity by sterically blocking its binding sites on actin. This hypothesis is supported by results showing differences between cofilin-2 binding constants in the presence and absence of Ca^2+^. However, similar degrees of severing and filament depolymerization rates were observed in both activation states of the filament determined by the binding of Ca^2+^ to the Tn complex. Therefore, regulation of cofilin-2 functions does not seem to depend on the position of Tpm-Tn on the filament.

Protection of the thin filament by Tpm3.12-R91C from disassembly by cofilin-2 was also observed in the presence of Tn, which demonstrated that the p.R91C variant interfered with the maintenance of the length of the reconstituted thin filaments, increasing their length. A similar effect was observed in a previous study in which Tpm3.12-R91C abnormally regulated tropomodulin, another protein that controls the length of the thin filaments. In the presence of Tpm3.12-R91C, the ability of tropomodulin to inhibit actin polymerization decreased, resulting in faster polymerization at the pointed end [13]. Since cofilin-2 localizes to the center of the sarcomere [22], defects in the regulation of cofilin-2 and tropomodulin caused by the substitution p.R91C may result in abnormal trimming of the thin filament near the pointed end.

It is worth mentioning that cofilin activity is pH-dependent [44,45]. Our in vitro assays were previously optimized to pH 7.5 [19]. As such, the observed effects in vivo can be either enhanced or diminished by pH fluctuations. This is important, especially since muscle fatigue causes a significant decrease of pH in myofibers [46,47], which may be a significant factor in affecting myopathic phenotype severity. Therefore, more detailed studies are required to determine the combined effects of different regulatory proteins and cellular conditions on cofilin-2 activity.

## 4. Materials and Methods

### 4.1. Muscle Protein Preparation

Actin (UniProt P68135) was isolated from rabbit pectoral muscle and purified according to the method described by Spudich [48]. An extinction coefficient of 0.63 mg mL^−1^ cm^−1^ at 290 nm and a molecular weight (MW) of 42,000 Da were used to determine the concentration of G-actin. The Tn complex was isolated from ether powder of the back and hind-limb rabbit skeletal muscle, as described by Potter [49]. The concentration of Tn was determined using the extinction coefficient of 0.45 at 280 nm (0.1%) and a MW of 69,000 Da.

### 4.2. Preparation of Recombinant Tropomyosin Tpm3.12 Variants and Cofilin-2

Construction of plasmids carrying cDNA encoding the wild-type human Tpm3.12 (UniProt P06753-1) and myopathy-mutant Tpm3.12-R91C and protein expression in bacterial cells were achieved as previously described [13]. Both Tpm variants had two amino acid N-terminal extensions (Ala-Ser) to compensate for the lack of acetylation of bacterially expressed proteins [50]. In brief, BL21 (DE3) cells (Novagen Inc., Madison, WI, USA) were transformed with pET11a plasmid (Novagen Inc., Madison, WI, USA) carrying cDNA sequences that translated into human Tpm3.12 or Tpm3.12-R91C. Cells were grown in Luria–Bertani (LB) medium with shaking until the culture reached 0.2 OD^550^, with expression induced with using isopropylthio-β-galactoside (IPTG). At 1.5 OD, cells were harvested, sonicated, and centrifuged for 1 h at 40,000 rpm at 4 °C. Tpm was separated from other proteins in two steps, with (NH_4_)_2_SO_4_ precipitation at 35–70% saturation, followed by ion exchange chromatography on Macro-Prep 25 Q resin (BioRad, Hercules, CA, USA) equilibrated to 20 mM Tris-HCl, pH 7.5, 0.5 mM DTT, 0.02% NaN_3_ using the NGC FPLC chromatography apparatus (Bio-Rad, Hercules, CA, USA). The protein was eluted from the column with a 0–1.0 M NaCl gradient and dialyzed against 10 mM Tris-HCl, pH 7.5, 30 mM NaCl, 2 mM MgCl_2_, 1 mM DTT, 0.02% NaN_3_. The concentration of isolated proteins was determined spectrophotometrically at 280 nm using the theoretical molar extinction coefficient of 17,880 M^−1^ cm^−1^. The coefficient was computed from the human amino acid sequence of Tpm3.12 (UniProt P06753-1) using the Expasy tool available at https://web.expasy.org/protparam/ (accessed on 10 November 2023).

Cofilin-2 (UniProt P45591) was expressed in BL21 (DE3) cells and purified as described in [32]. Expressionplasmid encoding murine GST-tagged cofilin-2 (pPL93) was gifted by Pekka Lappalainen, Helsinki University (Helsinki, Finland). In brief, cells harvested after induction of protein expression with IPTG were washed with 50 mM Tris-HCl (pH 7.5) and 150 mM NaCl and sonicated and centrifuged for 1 h at 40,000 rpm at 4 °C. The supernatant was mixed with glutathione-agarose beads (Pierce, Rockford, IL, USA), pre-equilibrated with buffer, as above, for one to two hours at 4 °C. Overnight digestion (4 °C) by human serum thrombin (Sigma-Aldrich, St Louis, MO, USA) was applied to cleave cofilin-2 from GST-bound beads. The cleaved protein was recovered by centrifugation and syringe filtration. The concentration of the protein was determined spectrophotometrically using the extinction coefficient of 0.79 mg mL^−1^ cm^−1^ at 280 nm and a MW 18,500 Da.

### 4.3. Cosedimentation Assay

Actin-cofilin-2 binding affinity was obtained by measuring the amount of cofilin-2 that cosediments with actin filaments covered by Tpm variants alone or by Tpm-Tn complex in the absence or presence of Ca^2+^ and was performed as described previously [19]. For cofilin-2 binding experiments, 3 μM F-actin saturated with either 2 μM Tpm3.12 or Tpm3.12-R91C, alone in the absence or presence of 3 μM Tn complex, was mixed with cofilin-2 at concentrations increasing from 0 to 10 μM in binding buffer (5 mM imidazole, pH 7.5, 1 mM DTT, 100 mM NaCl, and 2 mM MgCl_2_ and either 0.1 mM CaCl_2_ or 0.2 mM EGTA). After mixing, the proteins were incubated for 20 min at room temperature and then pelleted. The supernatants and pellets were separated on SDS–polyacrylamide gel electrophoresis (SDS-PAGE), and the densities of the protein bands were analyzed by the Image Lab software, version 6.0.1 (BioRad, Hercules, CA, USA). The concentration of free, unbound cofilin-2 was obtained from the supernatants, while the amount of cofilin-2 bound to F-actin was quantified from the pellets. Experimental data were fit to the Hill equation, to obtain the K_app_ and Hill cooperativity coefficient (α^H^), and plotted using SigmaPlot 12.5 (Systat Software Inc., San Jose, CA, USA), as described in [19].

Cofilin-induced dissociation of Tpm from the filament was obtained from the SDS gels used to separate proteins collected in pellets at increasing cofilin-2 concentrations. Tpm/actin densitometric ratios obtained at each cofilin-2 concentration were divided by the maximal value in each experiment and were drawn versus cofilin-2/actin molar ratio. The experimental points were fit to an exponential decay equation or linear regression in SigmaPlot 12.5 (Systat Software Inc., San Jose, CA, USA). The statistical significance of differences between groups was determined by one-way analysis of variance (ANOVA).

### 4.4. In Vitro Severing/Depolymerization Assay

Severing and depolymerization rates of actin filaments labeled in Gln41 with tetramethylrhodamine cadaverine (TRC) (Zedira, Darmstadt, Germany) by cofilin-2 were observed directly using an Olympus IX83 inverted fluorescence microscope (magnification 100×). Labeling of actin Gln41 with TRC was performed using bacterial transglutaminase as before [19]. The filaments were observed in a flow cell constructed by connecting two coverslips (24 × 24 mm and 32 × 24 mm) with parafilm strips to form flow channels. The channels were coated with positively charged lipids: DPPC (1,2-dipamitoyl-sn-glycero-3-phosphocholine; Sigma-Aldrich, St Louis, MO, USA) and TAP (1,2-dipalmitoyl-3-trimethylammonium-propane; Sigma Aldrich) [34], with a DPPC:TAP weight ratio of 9:1. After dispersing in Milli-Q water (2 mg/mL), the lipids were diluted in 5 mM of MgCl_2_ to 0.5 mg/mL and sonicated in a bath sonicator for one minute. An aliquot (50 μL) of lipids was introduced into a flow cell and incubated for one hour at 36 °C in a humid environment. The flow cells were rinsed with 25 mM Tris-HCl, pH 7.5, 25 mM KCl, 2 mM MgCl_2_ to remove excess.

After coating, each channel was washed with blocking buffer (25 mM Tris-HCl, pH 7.5, 25 mM KCl, 2 mM MgCl_2_ and 10 mg/mL of BSA) and then 240 nM bare TRC-actin or TRC-actin filaments pre-incubated with 80 nM Tpm variants ± 120 nM Tn were added. The unbound filaments were washed with buffer supplemented with antioxidant enzymes. When the Tpm-Tn complex was present, the blocking buffer was additionally supplemented with either 0.1 mM CaCl_2_ or 0.2 mM EGTA. The severing/depolymerization was started by addition of 240 nM cofilin-2 to the channel. Selected filaments were examined for the number of breaks caused by cofilin-2 and the length of the longest fragment obtained after severing using the cellSens Dimension software (Olympus Life Science, Tokyo, Japan).

## 5. Conclusions

Maintaining the length of thin filaments is critical for sarcomere structure and optimization of contractile force. Therefore, defects in actin turnover mechanisms may contribute to the development of muscle weakness in patients with congenital myopathy. Since Tn determines the binding of Tpm to actin with high affinity and regulates the position of Tpm on the filament in striated muscle, Tn should be included in functional analyses.

Our data indicates that the Tpm3.12-Tn complex inhibits, but does not abolish, the ability of cofilin-2 to sever and depolymerize actin filaments. The Tpm3.12-Tn complex is not removed from the filament, suggesting that cofilin-2 can sever and depolymerize the thin filament without compromising the ability of regulatory proteins to control contraction. Since cofilin-2 activity is on the same level in the presence and absence of Ca^2+^, the length of the thin filaments is maintained in the active and resting muscle fibers.

The myopathy-causing Tpm3.12-R91C variant increases the ability of Tpm3.12 to inhibit cofilin-2 activity, supporting the hypothesis that the phenotype typical of congenital myopathies, such as muscle weakness and disarray of the thin filaments, may result from both impaired Ca^2+^-dependent regulation of actin–myosin interactions and disturbances in regulation of cofilin-2-dependent maintenance of thin filament length. Since cofilin-2 is only one of several proteins involved in actin length regulation, further studies are needed to fully decipher the mechanism of thin filament length maintenance and the impact of pathological Tpm variants on this process.

## Figures and Tables

**Table 1:** Parameters of cofilin-2 binding to actin filaments regulated by Tpm3.12 variants and the troponin complex.

	−Tn		+Tn (+Ca^2+^)		+Tn (−Ca^2+^)	
	K_app_ [μM^−1^]	α^H^	K_app_ [μM^−1^]	α^H^	K_app_ [μM^−1^]	α^H^
F-actin	6.0 ± 0.1	2.5 ± 0.1	n.a.	n.a.	n.a.	n.a.
F-actin-Tpm3.12	6.6 ± 0.2	1.2 ± 0.4	5.5 ± 0.3 *	3.2 ± 0.5	12.5 ± 1.8 *	1.5 ± 0.3
F-actin-Tpm3.12-R91C	5.2 ± 0.2	1.7 ± 0.2	3.5 ± 0.4 *	2.1 ± 0.3	10.5 ± 0.8 *	1.4 ± 0.2

The parameters were obtained by fitting the experimental points shown in Figure 1 to the Hill equation. All numbers are the mean ± standard error (SE) obtained from the curve fit parameters computed in SigmaPlot 12.0. * Statistically significant differences in K_app_ obtained for cofilin-2 binding in the presence and absence of Ca^2+^ (*p* < 0.05).

## Data Availability

The raw datasets generated during the investigation and/or analyzed throughout this paper are publicly available at the RepOD repository website: https://doi.org/10.18150/ZDLMTM (accessed on 10 November 2023).
